# SPINDLY, ERECTA, and Its Ligand STOMAGEN Have a Role in Redox-Mediated Cortex Proliferation in the *Arabidopsis* Root

**DOI:** 10.1093/mp/ssu106

**Published:** 2014-09-29

**Authors:** Hongchang Cui, Danyu Kong, Pengcheng Wei, Yueling Hao, Keiko U. Torii, Jin Suk Lee, Jie Li

**Affiliations:** ^a^Department of Biological Science, Florida State University, Tallahassee, FL 32306–4295, USA; ^b^ Present address: Biotechnical Group, Institute of Rice Research, Anhui Agricultural Academy of Science, 40#, Nongke South Road, Hefei, Anhui, 230031, China; ^c^Howard Hughes Medical Institute, Department of Biology, University of Washington, Seattle, WA 98195-1800, USA

**Keywords:** SPY, ERECTA, STOMAGEN, redox homeostasis, ROS signaling, abiotic stress, cortex proliferation, *Arabidopsis thaliana*.

## Abstract

Reactive oxygen species (ROS) are harmful to all living organisms and therefore they must be removed to ensure normal growth and development. ROS are also signaling molecules, but so far little is known about the mechanisms of ROS perception and developmental response in plants. We here report that hydrogen peroxide induces cortex proliferation in the *Arabidopsis* root and that SPINDLY (SPY), an O-linked glucosamine acetyltransferase, regulates cortex proliferation by maintaining cellular redox homeostasis. We also found that mutation in the leucine-rich receptor kinase ERECTA and its putative peptide ligand STOMAGEN block the effect of hydrogen peroxide on root cortex proliferation. However, ERECTA and STOMAGEN are expressed in the vascular tissue, whereas extra cortex cells are produced from the endodermis, suggesting the involvement of intercellular signaling. *SPY* appears to act downstream of *ERECTA*, because the *spy* mutation still caused cortex proliferation in the *erecta* mutant background. We therefore have not only gained insight into the mechanism by which SPY regulates root development but also uncovered a novel pathway for ROS signaling in plants. The importance of redox-mediated cortex proliferation as a protective mechanism against oxidative stress is also discussed.

## SUMMARY

Reactive oxygen species (ROS) are signaling molecules, but how they are perceived in plants remains unclear. This study showed that cortex proliferation in the *Arabidopsis* root can be induced by hydrogen peroxide and that the receptor kinase ERECTA and one of its ligands, STOMAGEN, are involved in a signaling pathway that couples ROS sensing with redox-mediated cortex proliferation. This study also revealed a new role for SPINDLY (SPY), a putative O-GlcNAc transferase, in cellular redox homeostasis.

## INTRODUCTION

Reactive oxygen species (ROS), such as singlet oxygen (^1^O_2_), superoxide anion (O^·^_2_^–^), hydroxyl radical (HO^·^), and hydrogen peroxide (H_2_O_2_), are produced in all aerobic organisms as by-products of the metabolic processes in mitochondria and peroxisomes (Blokhina and Fagerstedt, 2010). In plants, the chloroplast is another major site of ROS production (Mullineaux and Baker, 2010). H_2_O_2_ can also be generated at the cell surface directly through the activity of plasma-membrane-bound NADPH oxidases (Sagi and Fluhr, 2006). Although accumulation of ROS is insignificant under optimal growth conditions, their production is increased under various stresses, biotic and abiotic, and cellular ROS level can build up to high levels (Miller et al., 2009).

ROS are highly reactive—they can oxidize nearly all major biologically active molecules, including lipid, protein, and nucleic acids, causing damage to the cellular membrane system, inactivation of enzymes and cellular structures, and mutation in DNA. At high concentrations, ROS become lethal. To avoid these deleterious effects, cells must be able to control the cellular level of ROS tightly. In both plants and animals, a complex antioxidant system has evolved for detoxification of ROS (Wormuth et al., 2007). The first layer of this defense is formed by small reducing compounds, such as ascorbic acids and glutaredoxin. The second comprises enzymes that either convert ROS to water or are responsible for regeneration of the small antioxidant molecules. Examples of the first group of enzymes include superoxide dismutase, peroxidase, and catalase, whereas ascorbate reductase and glutathione reductase belong to the latter group. A third level of defense is the induction of transcriptional regulators that coordinate the relocation of resources from the developmental program to stress responses and survival. When ROS production exceeds the capacity of the antioxidant system or when the ROS detoxifying system is compromised, ROS accumulates and this could cause cell damage or even death.

To maintain redox homeostasis requires close monitoring of cellular ROS. In bacteria, cellular redox status is monitored and regulated by proteins whose activity depends on oxidation state (Green and Paget, 2004). This ancient redox-sensing system probably remains functional in the chloroplasts and mitochondria, which are derived from bacteria through endosymbiosis (Foyer and Noctor, 2003). Mounting evidence indicates that ROS is also part of the retrograde signals from mitochondria or chloroplasts that activate nuclear gene expression in response to various stresses. H_2_O_2_, however, is the only form that can act as a signaling molecule in the communication between cells or cellular compartments, because it is the most stable type of ROS and can move across the membrane system, a prerequisite for inter-organelle or intercellular signaling. In animals and plants, communication between cells or subcellular compartments involves a complex system. Nuclear proteins with a role in ROS response have been identified whose activity is also regulated by oxidation and reduction (Tron et al., 2002; Mukherjee and Burglin, 2006; Comelli and Gonzalez, 2007), but a MAP kinase cascade is involved as well (Grant et al., 2000).

H_2_O_2_ not only acts as a primary ROS signal but is also produced as a second messenger in many important biological processes. During incompatible host–pathogen interaction, ROS formed during the initial stage of infection activate membrane-bound NADPH oxidases RBOH D and F, resulting in a burst of H_2_O_2_ (Torres et al., 2002). The second wave of ROS formation is the one responsible for the hypersensitivity response, whereby local programmed cell death is induced to prevent pathogen spreading (Torres et al., 2006). In addition to their role in disease resistance, ROS are involved in many developmental processes, such as root-hair formation (Foreman et al., 2003), xylem differentiation (Ros Barcelo, 2005), and Casparian strip formation (Lee et al., 2013). ROS maximum occurs at the root apical meristem as well (De Tullio et al., 2010), but it appears to inhibit, rather than promote, root meristematic activity (Tsukagoshi et al., 2010). An accumulating body of evidence suggests that maintaining a proper redox status is important for normal plant growth and development. When the *ROOT MERISTEMLESS 1* gene is mutated, glutathione synthesis is disrupted, halting root growth soon after embryogenesis (Vernoux et al., 2000; Reichheld et al., 2007). Redox homeostasis is also essential for anther development (Xing and Zachgo, 2008; Hu et al., 2011), petal patterning (Hepworth et al., 2005), and plant growth (Pasternak et al., 2008).

How H_2_O_2_ is perceived in plants is still unclear, although a number of factors are known to play important roles in ROS signal transduction. One of the early responsive proteins is OXIDATIVE STRESS INDUCIBLE 1 (OXI1) (Rentel et al., 2004), which relays the ROS signal by phosphorylating MAPK3, MAPK4, and MAPK6 (Moon et al., 2003). These kinases in turn activate genes that are involved in ROS response (Moon et al., 2003). Some evidence indicates that MEKK1 also acts upstream of MAPK3 and MAPK6 in ROS signaling (Nakagami et al., 2006). In addition, a number of proteins have been reported to play a role in orchestrating plant development with ROS homeostasis or response, such as RCD1 and UBP1 in root apical meristem (Teotia and Lamb, 2010; Tsukagoshi et al., 2010), PERIANTHIA (PAN) in flowering patterning (Hepworth et al., 2005), OsMADS3 in stamen development (Hu et al., 2011), and ROXY1 and ROXY2 in anther development (Xing and Zachgo, 2008).

SPINDLY (SPY) is an O-linked N-acetyl glucosamine (GlcNAc) transferase (Olszewski et al., 2010). The *spy* mutant was initially identified from a screen for mutations that relieve the germination-inhibitory effect of paclobutrazol, a GA biosynthesis inhibitor (Jacobsen and Olszewski, 1993). Because most of the *spy* mutant phenotypes can be reproduced by exogenous application of GA and because GA biosynthesis per se is not affected in the *spy* mutant, SPY was thought to be a repressor of GA signaling (Jacobsen et al., 1996). The *spy* mutant, however, has pleiotropic defects such as altered phylotaxy, male sterility, early flowering, and a spindly shoot (hence its name) (Jacobsen and Olszewski, 1993), but not all these developmental defects can be phenocopied by exogenous GA application (Swain et al., 2001). Some features of the *spy* phenotype, such as smaller leaves, are even the opposite of what is expected when plants experience an elevated level of GA signaling. These observations have led to the finding that SPY is also involved in cytokinin signaling (Greenboim-Wainberg et al., 2005), BR signaling (Shimada et al., 2006), light signaling, and circadian rhythms (Tseng et al., 2004). A recent study showed that SPY also regulates drought tolerance, and this role does not appear to involve GA signaling (Qin et al., 2011).

The *spy* mutation also causes developmental defects in the root—on hard medium the root becomes less wavy (Swain et al., 2002), and root growth is less sensitive to cytokinin inhibition (Greenboim-Wainberg et al., 2005). Recently, we found that SPY also plays a role in root cortex proliferation (Cui and Benfey, 2009b). In wild-type primary root, middle cortex is not produced until at least 7 d after germination (Supplemental Figure 1), whereas, in *spy-3* root, middle-cortex formation occurs as early as 3 d after germination (Cui and Benfey, 2009b). The physiological significance of cortex proliferation and how SPY regulates middle-cortex formation are still unclear. Through transcriptome analysis, we found that SPY plays a role cellular redox homeostasis and that this role is critical for its function in regulating cortex proliferation. Unexpectedly, we also found that the leucine-rich receptor kinase ERECTA and its putative ligand STOMAGEN are required for redox-mediated cortex proliferation.

## RESULTS

### Genome-Wide Identification of Genes Affected in the *spy* Mutant Root

To elucidate the mechanism by which SPY regulates root development, we first identified the genes affected by the *spy* mutation in the *Arabidopsis* root by transcriptomic analysis. One-week-old wild-type (Columbia, Col) and *spy-3* seedlings were compared using the Affymetrix ATH1 whole-genome microarray. With a threshold of 1.5-fold change and a false discovery rate of 0.01, we identified 106 genes as down-regulated (with a lower level of transcripts) and 26 genes as up-regulated (with a higher level of transcripts) in *spy-3* (Supplemental Table 1). To reveal the biological processes in which SPY is involved, we then conducted Gene Ontology (GO) analysis with the genes affected by the *spy* mutation. Surprisingly, this analysis showed that a large fraction of genes have no known functions (Supplemental Figure 2), suggesting that SPY is involved in processes other than GA and cytokinin signaling.

### SPY Has a Role in Cellular Redox Homeostasis

Among the genes whose expression level is altered by the *spy* mutation, many genes are associated with stress response. Using the AmiGO program, which is a GO term-enrichment tool (http://amigo.geneontology.org/cgi-bin/amigo/go.cgi), we further found that genes involved in redox homeostasis were significantly over-represented (*p* < 10^–5^; Supplemental Figure 3), which suggests that SPY may have a role in regulating the cellular redox status. Of the 106 genes down-regulated in the *spy*-3 mutant, for example, more than 10% are associated with redox homeostasis, including six peroxidases, two dehydroascorbate reductases, one catalase, one oxidase, and one reactive-oxygen-burst-homology gene (*ROBHD*) (Supplemental Table 1). Peroxidases are a diverse group of enzymes, because some catalyze the formation of hydrogen peroxide, others consume hydrogen peroxide in lignin biosynthesis (Lee et al., 2013). In addition, *LBD41* (Licausi et al., 2011) and AT3G16770, a member of the plant-specific *ERF/AP2* transcription factor family, are known to be involved in oxidative stress response (Ogawa et al., 2005) (Supplemental Table 1). Among the up-regulated genes, At1g28480, a glutaredoxin-family protein, and At4g34410, also known as *REDOX RESPONSIVE TRANSCRIPTION FACTOR 1* (*RRTF1*), are apparently involved in oxidative stress response (Khandelwal et al., 2008). Moreover, the only over-represented GO category in the up-regulated genes is associated with response to jasmonic acid (1.2e-4), which has been shown to antagonize ROS in lignin biosynthesis (Denness et al., 2011) and thus could also play a role in redox homeostasis.

**Figure 1:**
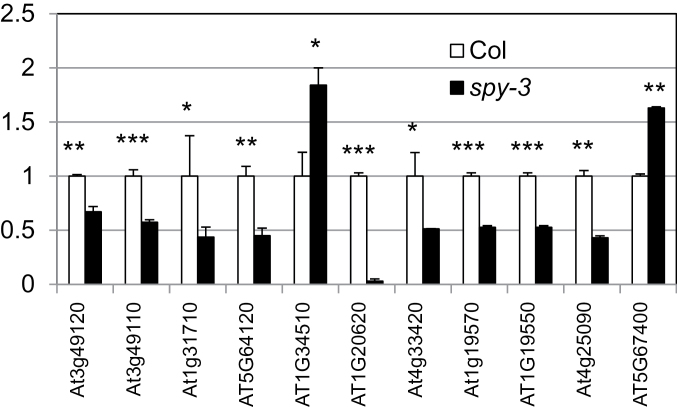
Quantitative RT–PCR Assay, Showing Altered Transcript Levels of Genes Involved in Redox Homeostasis in *spy-3* Root. At3g49120 (PERX33), At3g49110 (PERX34), At5g64120, At1g34510, At4g33420, and At5g67400 (PERX73) are peroxidases; At1g31710 is a copper amine oxidase; At1g20620 (CAT3) is a catalase; At1g19570 and At1g19550 are dehydroascorbate reductases; At4g25090 is a reactive oxygen-burst homolog. These genes were selected because microarray data showed that their expression level was altered by the *spy* mutation. The asterisks indicate the significance of the change by *t*-test. * *p* < 0.05; ** *p* < 0.01; *** *p* < 0.001.

**Figure 2:**
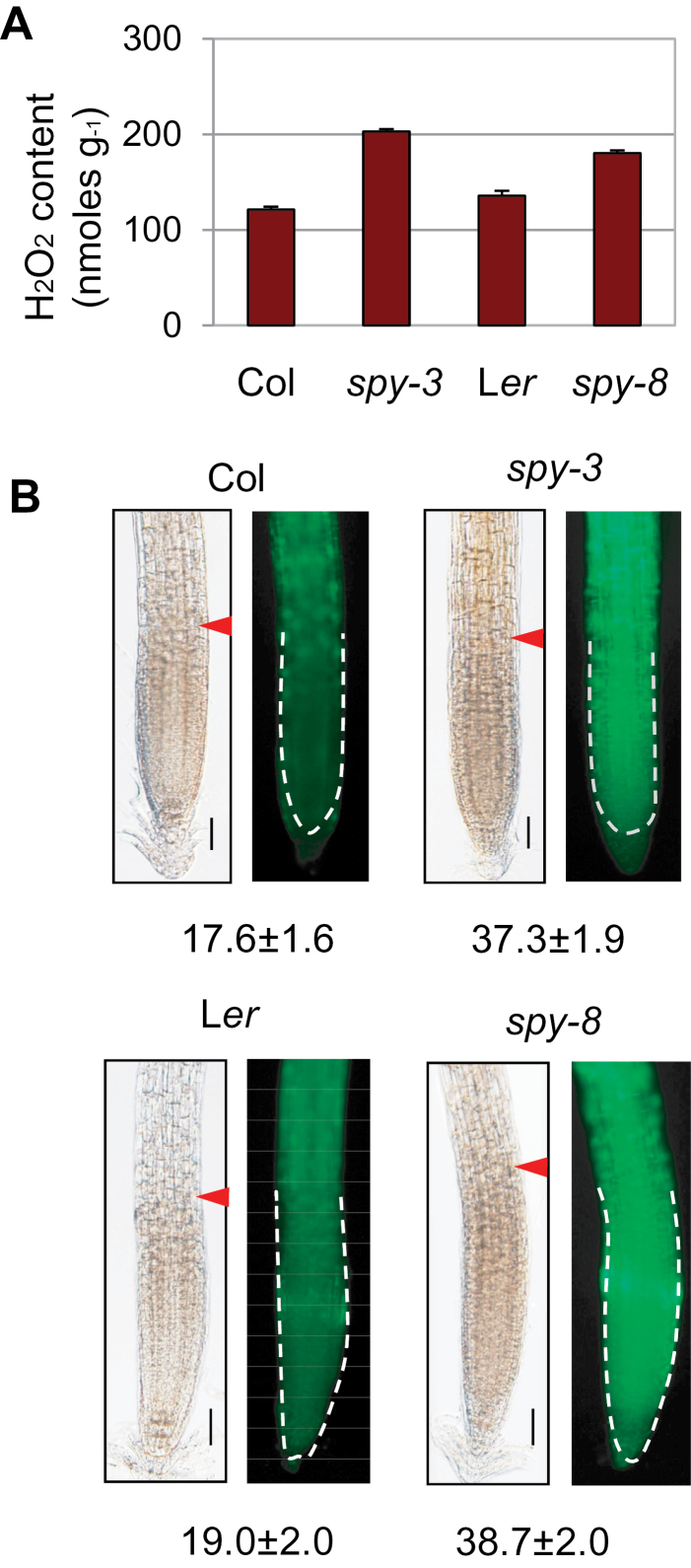
Hydrogen Peroxide Assays Showing Elevated ROS Level in the *spy* Mutants. **(A)** Quantitative assay of H_2_O_2_ in the roots of 1-week-old seedlings grown in MS medium. The error bars represent standard deviation from triplicate measurements. The differences are highly significant (*p* < 0.001 or 0.01 for the Col versus *spy-3* and L*er* versus *spy-8* comparisons, respectively; *t*-test). **(B, C)** Detection of H_2_O_2_ with dichloroflurescin diacetate. The numbers below different genotypes are the fluorescence intensity in the meristem zone (mean ± standard deviation, *N* = 15), as marked by arrowhead (left) and also outlined by broken line (right). Bars = 50 μm.

To determine whether SPY has a role in redox homeostasis, we examined the transcript level of the 11 oxidative-stress-related genes in Col and *spy-3* root by quantitative RT–PCR. As shown in Figure 1, all but two had reduced transcript levels in the *spy* mutant. We next compared the level of H_2_O_2_ because this ROS species is much more stable than others and can be more reliably quantified with a commercial kit (see ‘Methods’ section). Using this quantitative assay, we showed that *spy-3* root had an elevated level of H_2_O_2_ (Figure 2A). Although the increase seems small, it is significant and agrees with a recent study using a different assay for H_2_O_2_ (Achard et al., 2008). To determine whether this is specific to the *spy-3* allele, we performed the H_2_O_2_ assay with *spy-8*, which is in the L*er* background. As shown in Figure 2A, the *spy-8* mutant root had a higher level of H_2_O_2_ as well. These results demonstrate an important role for SPY in redox homeostasis.

Because middle cortex occurs in the meristem and elongation zone, we next compared H_2_O_2_ level in the root tips of *spy* mutants and wild-type seedlings using dichlorofluorescin diacetate, which is non-fluorescent dye but becomes fluorescent inside the cells after oxidization. As shown in Figure 2B, both *spy-3* and *spy-8* have a significantly higher level of fluorescence, indicating elevated levels of H_2_O_2_ (*p*-values are 3.25e-14 for the *spy-3* versus Col comparison and 2.43e-10 for the *spy-8* vs L*er* comparison. *t*-test, *N* = 15).

### SPY Represses Cortex Proliferation by Maintaining Cellular Redox Homeostasis

**Figure 3:**
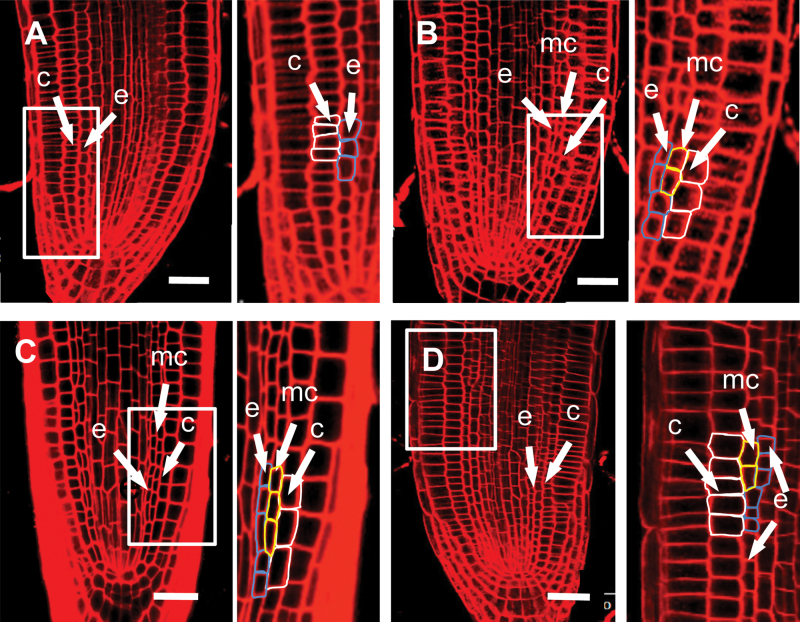
Middle-Cortex Formation Is Regulated by Cellular Redox Status. Confocal-microscopy images of wild-type (Col) **(A, B)** and *spy-3* roots **(C, D)**, 24h after transfer into water (A, C), 1mM H_2_O_2_ (B), or 1mM glutathione (D). The framed areas are shown on the right at a higher magnification. mc, middle cortex; c, cortex; e, endodermis. Bars = 20 μm.

The elevated level of ROS in the *spy* mutant raises the possibility that cortex proliferation might be a developmental response to oxidative stress. To test this hypothesis, we treated wild-type roots with hydrogen peroxide and examined the radial pattern by confocal microscopy. Five-day-old seedlings grown on MS medium were used for the experiment, because, at this stage, middle cortex has not formed (Cui and Benfey, 2009b). We did not observe cortex proliferation when H_2_O_2_ concentration was below 0.2mM or above 5mM, but all roots treated with 1–2mM H_2_O_2_ have produced at least one middle-cortex cell within 24h of H_2_O_2_ treatment (Figure 3B), whereas seedlings treated with water showed no sign of middle-cortex proliferation (Figure 3A).

The results described above lend strong support to the notion that the premature middle-cortex phenotype in the *spy* mutant is due to its elevated level of ROS. To test this further, we treated *spy-3* seedlings with 1mM glutathione, which is a biologically active antioxidant. Before or after treatment with water, roots of 5-day-old *spy-3* seedlings had continuous files of middle-cortex cells (Figure 3C) but, after 24h of glutathione treatment, most roots had no or very few middle-cortex cells in the root tip (Figure 3D). Glutathione had no effect on the middle cortex that had already formed before the treatment, which was visible in the upper part of the root (Figure 3D). These results suggest that cortex proliferation is regulated by cellular redox status and that SPY suppresses cortex proliferation by maintaining redox homeostasis.

### The Receptor Kinase ERECTA Is Required for Redox Sensing in Redox-Mediated Cortex Proliferation

**Figure 4:**
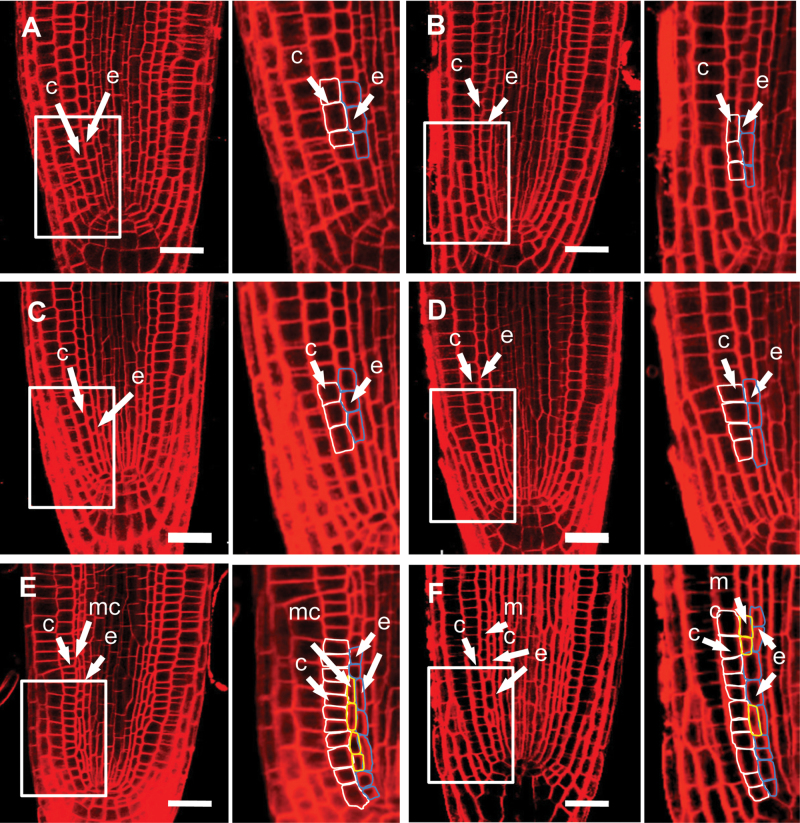
The Receptor Kinase ERECTA Is Required for Redox-Mediated Cortex Proliferation. Confocal microscopy images of wild-type (L*er*, **(A, B)**), *er-105* **(C, D)**, and *spy-8* roots **(E, F)**, 24h after transfer into water (A, C, E), 1mM H_2_O_2_ (B, D), or 1mM glutathione (F). c, cortex; mc, middle cortex; e, endodermis. Bars = 20 μm.

Because other *spy* alleles are in the L*er* background, we also treated L*er* seedlings with H_2_O_2_. Surprisingly, we observed no middle-cortex cells in the L*er* background even after prolonged treatment (48h) (Figure 4A and 4B). Because L*er* has a mutation in the receptor kinase ERECTA (Torii et al., 1996), this result suggests that ERECTA is required for ROS signaling that leads to cortex proliferation. However, the L*er* ecotype has other mutations, which could affect ROS signaling. We therefore examined the effect of H_2_O_2_ treatment on cortex proliferation in *er-105*, a well-characterized *ERECTA* null mutant in the Col background (Yokoyama et al., 1998; Shpak et al., 2004). Again, no middle-cortex formation was induced by H_2_O_2_ (Figure 4C and 4D). Based on these observations, we conclude that ERECTA plays a pivotal role in redox-mediated cortex proliferation.

### ERECTA Acts Non-Cell Autonomously in the Signaling Cascade Leading to Redox-Mediated Cortex Proliferation

**Figure 5:**
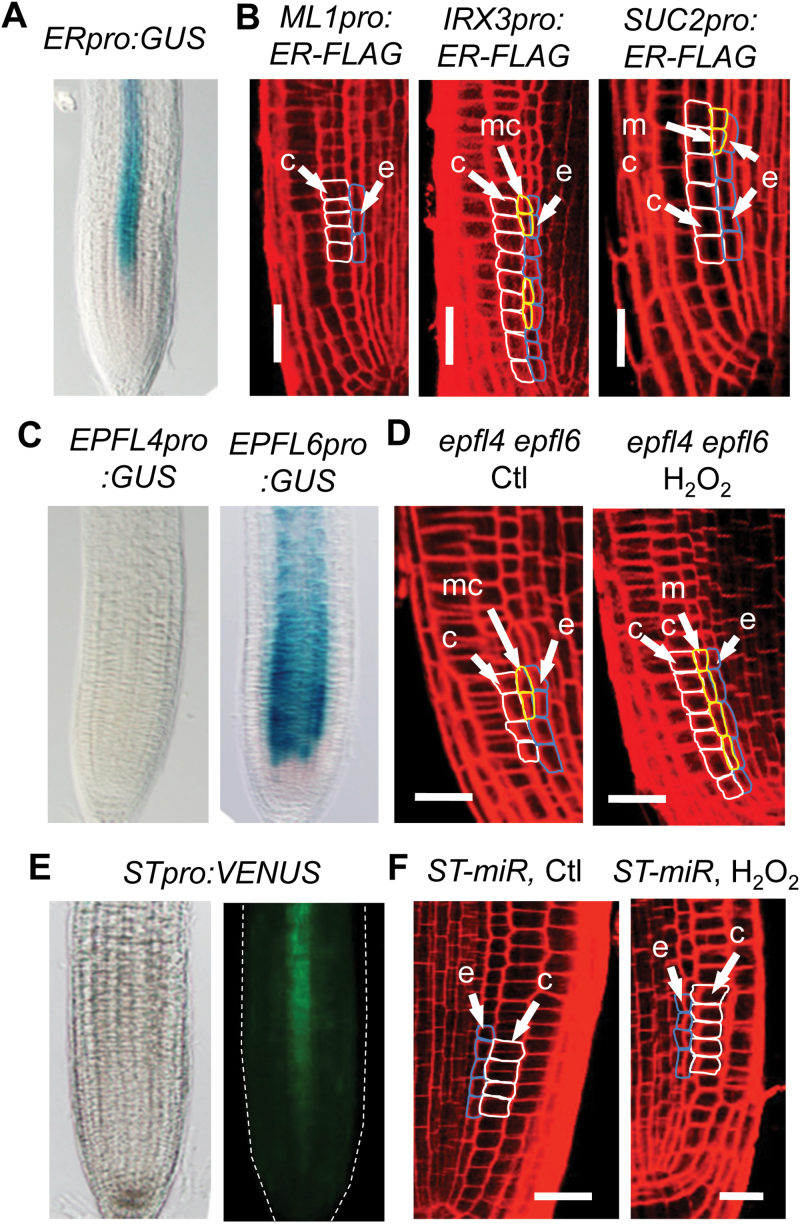
ERECTA and STOMAGEN Regulate Redox-Mediated Cortex Proliferation through Intercellular Signaling. **(A, C, E)** Expression pattern of ERECTA (A), EPFL4 ((C), left), EPFL6 ((C), right), and STOMAGEN ((E), bright field in left and fluorescence in the right), as shown by promoter–reporter transgenes. The reporter gene is GUS in (A) and (C), and VENUS in (E). **(B)** Root radial pattern in *er-105* mutant expressing ERECTA (ER) in the epidermis ((B), left), the xylem ((B), middle), or the phloem ((B), right) using the promoter of *ML1*, *IRX3*, or *SUC2*, respectively, after 24h of treatment with H_2_O_2_. **(D, F)** Root radial pattern in the *epfl4 epfl6* double mutant (D) or transgenic plants that contain an artificial miRNA targeting *STOMAGEN* (*ST-miR*) (F), after 24h of treatment with water (control) or H_2_O_2_. c, cortex; mc, middle cortex; e, endodermis. Bars = 20 μm.

A similar role for ERECTA in cortex proliferation in the inflorescence stem was recently reported, although the signal that induces cortex proliferation has yet to be identified (Uchida et al., 2012). In the stem, ERECTA is expressed in the vascular tissue and the epidermis (Uchida et al., 2012). However, ERECTA protein expressed in the phloem is sufficient to rescue the cortex proliferation defect in the *er* mutant, suggesting that cortex proliferation is induced by a signal coming from the vascular tissue (Uchida et al., 2012). To determine how ERECTA regulates ROS signaling in cortex proliferation, we first determined its expression pattern in the root by examination of GUS staining in transgenic plants that contain the *ERECTApro:GUS* transgene. As shown in Figure 5A, ERECTA was expressed in the vascular tissue, but not in the endodermis from which the middle cortex was derived. This result suggests intercellular communication is involved in redox-mediated cortex proliferation.

We next asked whether the phloem is also the cell type in which ERECTA regulates redox-mediated cortex proliferation in the root. To this end, we examined cortex response to H_2_O_2_ in transgenic plants that express a functional FLAG-tagged ERECTA fusion protein (ER-FLAG) in the *er-105* mutant background under the *SUC2*, *IRX3*, and *ML1* promoters. As in the shoot (Uchida et al., 2012), the promoters of *SUC2*, *IRX3*, and *ML1* used for the ERECTA expression in the root conferred specific expression in the phloem, xylem, and epidermis, respectively (Supplemental Figure 4). Unlike in the stem, however, H_2_O_2_ induced cortex proliferation in transgenic plants that express *ERECTA* in both the phloem and xylem (Figure 5B), and xylem-expressed *ERECTA* seemed to make the plants more responsive to H_2_O_2_ than that in the phloem, as indicated by a longer stretch of middle-cortex cells (Figure 5B). Based on these results, we think that *ERECTA* in the xylem plays a major role in redox-mediated cortex proliferation.

### STOMAGEN Is Likely the Ligand for ERECTA in Redox-Mediated Cortex Proliferation

In the inflorescence stem, cortex proliferation depends on not only ERECTA, but also its peptide ligands, EPFL4 and EPFL6 (Uchida et al., 2012). Because EPFL4 and EPFL6 are specifically expressed in the endodermis (Uchida et al., 2012), they are likely to be also the ligands for ERECTA in redox-mediated cortex proliferation in the root. To test this possibility, we first examined their expression pattern in the root by analyzing transgenic plants that carry the GUS reporter gene under the control of the *EPFL4* and *EPFL6* promoters. Although *EPFL6* was clearly expressed in the endodermis, *EPFL4* did not show any expression in the root (Figure 5C). Nevertheless, we cannot exclude the possibility that *EPFL4* is expressed at a low level in the root but plays a critical role in redox-mediated cortex proliferation. To determine the roles of EPFL4 and EPFL6 in root cortex proliferation, we therefore examined cortex response to H_2_O_2_ in the *epfl4 epfl6* double mutant. Surprisingly, the *epfl4 epfl6* double mutant showed normal response to H_2_O_2_ (Figure 5D). Under normal growth conditions, it even produced the middle cortex earlier than the wild-type.

*EPFL4* and *EPFL4* belong to a small family of genes encoding small peptides named Epidermal Patterning Factors (EPFLs) (Hara et al., 2009). One of the EPFLs, *STOMAGEN/EPFL9*, is expressed in the mesophyll cells and positively regulates stomata development in the epidermis (Kondo et al., 2010; Sugano et al., 2010). To determine whether this peptide plays a role in redox-mediated cortex proliferation, we first examined its expression pattern in the root by analyzing the *STOMAGEN:VENUS* reporter. Interestingly, *STOMAGEN* appeared to have a similar expression pattern to ERECTA—they are both expressed in the vascular tissue as early as the meristem zone (Figure 5E). We therefore next analyzed the cortex phenotype in plants that carry an artificial miRNA targeting the *STOMAGEN* gene (*ST-miR*), which has been shown previously to be able to efficiently reduce *STOMAGEN* expression (Sugano et al., 2010). Strikingly, cortex proliferation was not observed in the transgenic plants after treatment with 1mM H_2_O_2_ (Figure 5F), indicating that, like ERECTA, STOMAGEN is required for ROS-induced cortex cell proliferation.

### SPY Acts Downstream of ERECTA in Redox-Mediated Cortex Proliferation

Despite the requirement for ERECTA in redox-mediated cortex proliferation, middle cortex still occurs prematurely in *spy-8*, which is in the L*er* background (Cui and Benfey, 2009b) (Figure 4E). This is not specific to the *spy-8* allele, as other *spy* alleles in the L*er* background, such as *spy-12*, *spy-13*, *spy-15*, and *spy-17*, all had produced a middle cortex within a week after germination (Supplemental Figure 5). To determine whether the suppressive role of glutathione on cortex proliferation also depends on *ERECTA*, we treated *spy-8* roots with glutathione. However, no rescue of the middle-cortex phenotype was observed (Figure 4E and 4F). This result lends support to the notion that *ERECTA* is required for redox-mediated cortex proliferation. This result also suggests that *SPY* is epistatic to *ERECTA*.

**Figure 6:**
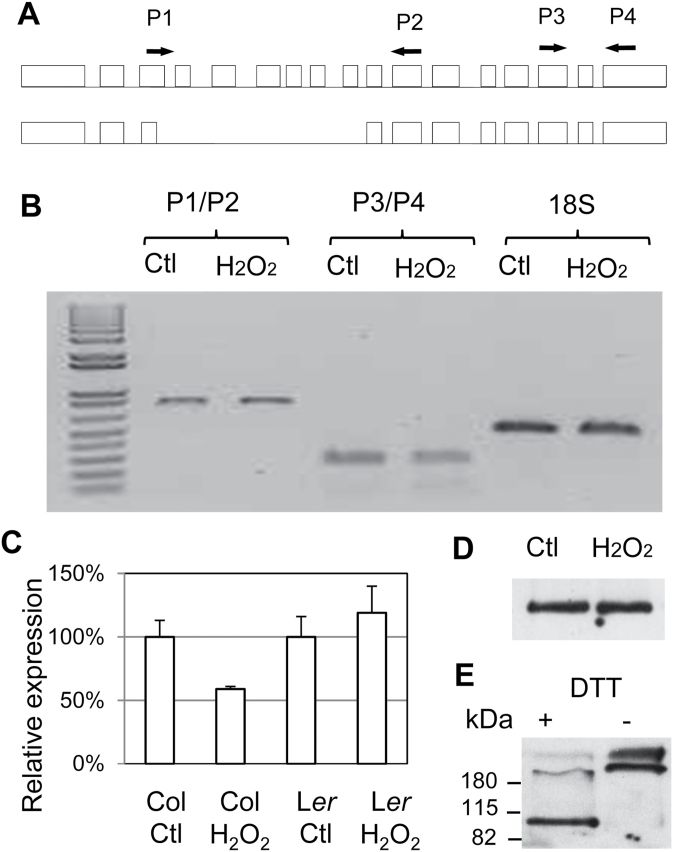
Effects on ROS on SPY Transcription, Alternative Splicing, and Protein Oxidization. **(A)** Diagram of alternative splicing of the SPY transcript, and the position of PCR primers for detection of the two isoforms. **(B)** RT–PCR assay of SPY transcript isoforms in the absence (Ctl) or presence of 1mM H_2_O_2_. The 18S rDNA was used as an internal control. The change is significant for Col (*p* < 0.05, *t*-test) but not for L*er*. **(C)** Relative *SPY* transcript level in Col and L*er* roots before and after treatment with water or 1mM H_2_O_2_ for 24h, as determined by real-time RT–PCR. The error bars represent standard deviations from triplicate measurements. **(D)** SPY–GFP protein level in roots after 24h of treatment with water and 1mM H_2_O_2_. Equal amounts of total protein extracts were loaded. **(E)** Western blot assay of the SPY–GFP protein in the presence (+) or absence of the reducing reagent dithiothreitol (DTT). The numbers on the left are the sizes of protein ladders.

ROS could induce middle-cortex formation in the Col background if *SPY* transcription were repressed, or if the *SPY* transcript were destabilized, or if the SPY protein were degraded or inactivated. An alternative form of *SPY* has been reported (Figure 6A), which lacks exons 4–8 and is most likely to be defunct, because mutations in several *spy* alleles fall in this region (Silverstone et al., 2007). It is therefore possible that H_2_O_2_ causes alternative splicing of the *SPY* transcript and accumulation of this truncated form of *SPY* transcript. To investigate this possibility, we designed primers that can distinguish the two transcripts by RT–PCR (717bp and 221bp). Our result, however, showed that the short transcript was not induced by H_2_O_2_ (Figure 6B).

We next asked whether SPY transcription is affected by ROS and whether ERECTA has a role in this regulation. To this end, we compared the *SPY* transcript level in Col and L*er* roots after 24h of treatment with water or 1mM H_2_O_2_. As shown in Figure 6C, *SPY* was slightly down-regulated in Col but remained unaltered in L*er*. However, the change in *SPY* transcript level in Col is small and may not cause dramatic change in *SPY* protein level. To address this, we examined the effect of H_2_O_2_ on SPY protein. We were unable to study the endogenous SPY protein because its concentration was too low to be detected by Western blot even after immunoprecipitation (Swain et al., 2001). Instead, we measured the SPY–GFP fusion protein expressed in the *spy-3* background under the *SPY* promoter (*SPYpro*:*SPY–GFP* in *spy-3*). As shown in (Figure 6D), the SPY–GFP protein level was similar after treatment by H_2_O_2_ or water. However, we noticed that the SPY–GFP protein was easily oxidized and oxidization induced oligomerization (Figure 6E). These results suggest that ROS promote cortex proliferation probably not by affecting SPY expression, but most likely by inactivating its enzymatic activity.

## DISCUSSION

Plants are sessile and therefore, to survive a precarious environment, they must be able to closely monitor and tightly maintain their cellular redox status, which can be disrupted by various stresses. Although ROS are known to act as signaling molecules, virtually nothing is known about the mechanism by which they are perceived and how this sensing mechanism is coordinated with the developmental program in plants. In this study, we showed that cortex proliferation in the *Arabidopsis* root is inducible by H_2_O_2_, which provides an example of positive developmental response to oxidative stress. We also showed that SPY has a role in maintaining cellular redox homeostasis and this role is mechanistically linked to its role in regulating cortex proliferation. Most importantly, we have uncovered a new redox signaling pathway that involves the receptor kinase ERECTA and its putative ligand STOMAGEN.

### SPY Suppresses Cortex Proliferation through Regulation of Cellular Redox Status

A role for SPY in redox homeostasis has been reported previously (Achard et al., 2008), but the underlying mechanism was not clear. In this study, we confirmed this finding using two independent assays for hydrogen peroxide. We further found that in the root ROS level is increased mainly in the apical meristem and the elongation zone, where the extra layer of cortex is formed. By transcriptomic analysis and RT–PCR assay, we showed that a significant number of genes that are involved in redox homeostasis are altered by the *spy* mutation, which provides a molecular basis for the role of SPY in cellular redox homeostasis.

Several pieces of evidence support the conclusion that SPY suppresses cortex proliferation by maintaining cellular redox homeostasis. First, H_2_O_2_ level was elevated in *spy*. Second, middle cortex is induced in the wild-type by exogenous H_2_O_2_. Third, *spy* mutant roots form a middle-cortex layer prematurely, and this layer is suppressed by glutathione, a reducing reagent. Because ROS are produced in the vascular tissue as an essential part of the xylem differentiation program (Jiang et al., 2012), it is likely that cortex proliferation under normal growth conditions is also a developmental response to oxidative stress.

In addition to *spy*, several other mutants have been shown to form middle cortex prematurely, such as *scr* and *lhp1* (Cui and Benfey, 2009b); the GA biosynthesis or signaling mutants *ga1*, *rga*, and *gid1* (Cui and Benfey, 2009b); and the ethylene signaling mutant *eto1* (Cui and Benfey, 2009a). There is evidence that GAI and RGA play a role in redox homeostasis (Achard et al., 2008); it is therefore likely that the cortex proliferation phenotypes in these mutants are caused by elevated levels of ROS as well, which warrants investigation.

### ERECTA and STOMAGEN Constitute a Novel ROS Signaling Pathway

Many components of the antioxidant system have been identified, but so far little is known about the early events of redox signaling (Potters et al., 2009). Our finding that ERETCA is required for redox-mediated cortex proliferation signifies the identification of a novel redox signaling pathway.

How is the redox signal perceived by ERECTA? The answer most likely lies in the findings that STOMAGEN, its putative ligand, is a cysteine-rich peptide. Structural studies have shown that the three-dimensional conformation of STOMAGEN can be modulated by its redox status (Kondo et al., 2010; Ohki et al., 2011). It is conceivable that, under oxidative stress, STOMAGEN is activated and the oxidized form in turn binds to ERECTA, thus initiating the signaling pathway.

Our finding that cortex proliferation occurs in the endodermis and that ERECTA and STOMAGEN are expressed in the vascular tissue suggests that intercellular signaling is involved in redox-mediated cortex proliferation. Although ERECTA is expressed in both the phloem and the xylem, the xylem-expressed protein seems to play a major role in redox signaling. ERECTA is also required for cortex proliferation in the inflorescence stem, but, unlike in the root, only the phloem-expressed protein was required for cortex proliferation (Uchida et al., 2012). In addition, the ligands for ERECTA signaling are also different. In the inflorescence stem, EPFL4 and EPFL6 are required for ERECTA in cortex proliferation (Uchida et al., 2012), whereas in the root STOMAGEN works together with ERECTA. These results suggest that either the signals that instruct cortex proliferation in the root and stem are distinct, or they are ROS but come from different sources. We are leaning towards the latter explanation because all EPFL peptides are cysteine-rich and therefore can sense ROS in a similar manner to that by STOMAGEN. Unlike EPFL4 and EPFL6, which are expressed in the endodermis, in roots STOMAGEN is expressed in the same tissue as ERECTA, the vascular tissue, suggesting that the as-yet unidentified intercellular signaling act non-cell autonomously to promote cortex cell divisions.

The question is, how does the STOMAGEN–ERECTA pair regulate cortex proliferation in the endodermis? Presently, we do not have an answer to this, but one possible mechanism is the activation of membrane-bound receptors that are specifically expressed in the endodermis and act downstream of the STOMAGEN–ERETCA signaling pathway. Another possibility is through the regulation of ROBHF. ROBHF is a NADPH oxidase that is expressed in the vascular tissue and is responsible for the production of ROS as an integral component of the xylem differentiation program (Jiang et al., 2012). When STOMAGEN is oxidized, activated ERECTA could enhance ROS production by increasing the enzymatic activity or the expression level of ROBHF. ROS then diffuse into the endodermis, causing inactivation of the SPY protein by oxidization and oligomerization. Interestingly, ROBHF gene expression is inducible by high salt and oxidative stress (Jiang et al., 2012), so the same mechanism could explain cortex proliferation under stress. More work is needed to identify the components in this ROS signaling pathway that effect cortex proliferation in the root.

ERECTA was first identified as a regulator of inflorescence growth (Redei, 1965; Torii et al. 1996). Subsequently, it was found to be involved in many other biological processes (van Zanten et al., 2009), including shoot growth and branching (Douglas et al., 2002), heat-stress response (Qi et al., 2004), disease resistance (Godiard et al., 2003), and stomatal patterning (Shpak et al., 2005). In the leaves, STOMAGEN is expressed in the mesophyll cells, whereas guard cells that form the stomata are produced in the epidermis (Kondo et al., 2010; Sugano et al., 2010). Other members of the EPF family of small peptides have been shown to directly bind to and act as ligands for ERECTA (Lee et al., 2012; Uchida et al., 2012). It is possible that ERECTA-mediated ROS signaling is the common mechanism underlying these biological processes.

### Interplay between SPY and the ERECTA–STOMAGEN Signaling Pathway in Redox-Mediated Cortex Proliferation

Although the ERECTA–STOMAGEN pair is required for redox signaling leading to cortex proliferation, mutation in *SPY* still causes cortex proliferation in the *erecta* mutant background, indicating that SPY acts downstream of the ERECTA–STOMAGEN signaling pathway. As shown in our *in vitro* experiment, the SPY protein is susceptible to oxidization. We therefore propose that at least one mechanism by which ROS induce cortex proliferation is through inactivation of the enzymatic activity of SPY. However, this is unlikely to be the only mechanism, as H_2_O_2_ treatment does not affect the radial patterning in the *erecta* mutant. One explanation is that the SPY protein is not as easily oxidized *in planta* as *in vitro*, and SPY is still active at the concentration of H_2_O_2_ used in the experiment. In view of our recent finding that epigenetic mechanisms are involved in cortex proliferation (Cui and Benfey, 2009b), it is more likely that the O-GlcNAc modification or other epigenetic marks that remain on target proteins are sufficient to block cell cycle progression. The inhibitory effect of O-GlcNAc modification on cell cycle regulators can also explain the requirement for the ERECTA–STOMAGEN signaling pathway, because protein phosphorylation as a result of the signal transduction act antagonistically with O-GlaNac in gene regulation (Wang et al., 2010).

**Figure 7:**
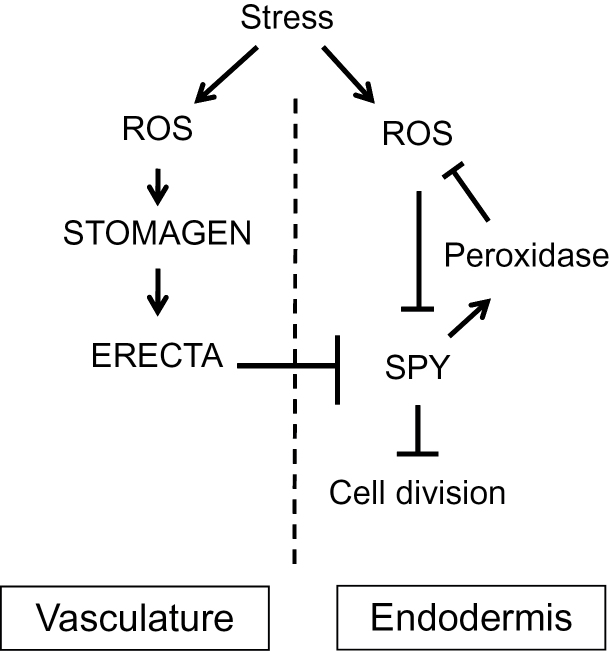
Schematic of the Interplay between SPY and the STOMAGEN–ERECTA Signaling Pathway in ROS Sensing and Redox-Mediated Cortex Proliferation in the *Arabidopsis* Root. Under stress, plants accumulate ROS, which oxidize and activate STOMAGEN. STOMAGEN in turn activates ERECTA, which exerts its effect on mitosis in the endodermis through intercellular signaling. ROS also oxidizes and inactivates SPY, which normally represses middle-cortex formation by maintaining the expression of peroxidases and thus cellular redox. SPY acts downstream of ERECTA, but how SPY is affected by the ERECTA signaling pathway remains unknown.

The interplay between SPY and the ERECTA–STOMAGEN signaling pathway is complex. As depicted in Figure 7, under normal growth conditions, SPY suppresses premature cortex proliferation by maintaining a relatively more reductive redox status, but under oxidative stress the SPY protein is inactivated by oxidization. ROS, particularly H_2_O_2_, also activate the ERECTA signaling pathway through oxidization of STOMAGEN, leading to induction of cortex proliferation. Because middle cortex is formed in the *spy* mutants in the L*er* background, SPY must act downstream of ERECTA. The interaction between SPY and ERETCA must be indirect, as ERECTA and STOMAGEN are expressed in distinct cell types. To elucidate the mechanism underpinning redox-mediated cortex proliferation, we need to first identify the factors that lie downstream of the ERECTA signaling pathway as well as the cell cycle genes that are involved in cortex cell proliferation, regulated by SPY and respond to oxidative stress, which will be pursued in future research.

### Cortex Proliferation Is Likely a Protective Mechanism against Oxidative Stress

In some plants, such as rice and maize, an air channel is formed within the cortex by localized cell death and dissolution of some cortex cells (He et al., 1994). This air channel, called aerenchyma, permits gas exchange between the root and the shoot and therefore ensures plant survival. Because ROS accumulates under hypoxia, aerenchyma formation is regarded as an adaptive response to oxidative stress (He et al., 1994). Our observation that middle cortex is induced by H_2_O_2_ suggests that cortex proliferation may be another protective developmental response to stresses. By increasing the number of cortex layers, plants would be able to restrict the entry of harmful elements such as salts and therefore maintain a healthy redox status in inner cells, which could in turn increase tolerance of salt and drought in the shoot. Future studies are needed to determine whether this is the case.

## METHODS

### Plant Materials and Treatments

The *spy* mutant alleles used in this study were described previously (Jacobsen and Olszewski, 1993; Silverstone et al., 2007). The *STpro:VENUS* and *ST-miRNA* transgenic lines were provided by Dr. Hara-Nishimura (Sugano et al., 2010). The following materials are generated in the Torii lab: *ERpro:GUS*, *ML1pro:GUS*, *IRX3:GUS*, *SUC2pro:GUS*, *ML1pro:ER-FLAG*, *IRX3pro:ER-FLAG* and *SUC2pro:ER-FLAG*, *EPFL4pro:GUS* and *EPFL6:GUS* (Uchida et al., 2012).

Unless specified, seedlings were grown in sterile conditions. For this purpose, seeds were surface-sterilized with 10% bleach plus 0.1% Tween 20, thoroughly washed with sterile water, and sown on MS medium in a square Petri dish (100mm × 100mm). For RNA preparation, the seeds were sown on a nylon mesh (400 mesh size) that was placed on MS medium. The plates were placed vertically in a Percival growth chamber under a 16-h light/8-h dark regime.

### Microarray Experiment and Analysis

Roots of 1-week-old wild-type and *spy-3* seedlings were collected and ground in liquid nitrogen. RNA isolation was performed with the Plant RNeasy Kit (Qiagen), and 2 μg of total RNA was used for cDNA synthesis with the Reverse Transcriptase III Kit (Invitrogen, USA). The cDNA was amplified and labeled with the kit from Affymetrix, which was followed by hybridization to the Affymetrix ATH1 whole-genome microarray by Expression Analysis Co. (Research Triangle Park, North Carolina, USA). For each sample, three biological replicates were done. The ANNOVA method for data analysis was used to identify genes differentially expressed between the wild-type and *spy* mutant according to Levesque et al. (2006).

### Hydrogen Peroxide Assays

Quantitative assay was performed using the Amplex® Red Hydrogen Peroxide assay kit (Cat. No. A22188, Invitrogen) according to the vendor’s instructions. For sample preparation, roots of 1-week-old seedlings grown on MS medium or leaves of 1-month-old plants grown in soil were first ground in liquid nitrogen. Three volumes of H_2_O were then added and the tissues were thoroughly mixed by vigorous vortexing. After centrifugation at 12 000rpm for 20min at 4ºC, the supernatants were assayed for H_2_O_2_. To prevent H_2_O_2_ degradation, all samples were analyzed immediately.

For the fluorescence-based assay, roots were incubated for 15min in 20 μM of dichlorofluorescin diacetate (D6883-50, Sigma, USA) in phosphate saline buffer (pH 7.3), rinsed twice in phosphate saline buffer, and examined at excitation 488nm and emission 535nm (GFP filter suits this purpose). Fluorescence intensity was quantified using the ImageJ program.

### Microscopy and Other Methods

GUS staining was performed according to the *Arabidopsis* lab manual (Weigel and Glazebrook, 2002). Bright field and GFP fluorescence imaging was performed with an Olympus BX61 compound microscope. For confocal microscopy, seedling roots were stained with FM-64, and images were taken with a Zeiss LSM510 confocal microscope.

To amplify the two isoforms of SPY transcript by RT–PCR, the following primers were used, which should yield a product of 717 or 221bp for the full-length or truncated transcript, respectively: RT_SPY_FW2, ACAATGCCTTGAGCTGCTACGA (in the third exon) and RT_SPY_RV2, TCATGGCAAGCAATCGGTTCTG (in the tenth exon).

Total RNA was extracted from 100mg tissues with the Plant RNeasy mini Kit (Qiagen) and 1 μg RNA was converted into cDNA with the SuperScript® III First-Strand Synthesis System (Invitrogen, USA). For quantitative RT–PCR, we used the ABI 7500 real-time PCR system and the PerfeCTa® qPCR FastMix® II kit (Quanta, USA). PCR cycling includes 30’’ denature at 94ºC, 30’’ annealing at 53ºC, and 30’’ extension at 65ºC.

To detect the SPY protein, the SPY–GFP fusion protein expressed under the SPY promoter in transgenic plants was first pulled down using a GFP antibody (Ab290, Abcam, UK). After heat denaturation in 2x sample buffer with or without DTT, the immunoprecipitates were resolved in 8% SDS–PAGE gel, transferred onto nitrocellulose membrane (Hybond-N+, GE, USA), and blotted using the same GFP antibody.

## SUPPLEMENTARY DATA

Supplementary Data are available at *Molecular Plant Online.*

## FUNDING

Funding for this work was from a set-up fund from the Florida State University (to H.C.). K.U.T. is an HHMI-GBMF investigator.

## Supplementary Material

Supplementary Data
