# Supplementary material for: SPINDLY, ERECTA, and Its Ligand STOMAGEN Have a Role in Redox-Mediated Cortex Proliferation in the Arabidopsis Root
Source: Mol Plant. 2014 Sep 29;7(12):1727–39. doi: 10.1093/mp/ssu106 (PMC4261839; doi:10.1093/mp/ssu106)
Supplement: Supplementary Data [file supp_7_12_1727__index.html]

SPINDLY, ERECTA and its ligand STOMAGEN have a role in Redox-mediated Cortex Proliferation in the Arabidopsis Root — SPINDLY, ERECTA and its ligand STOMAGEN have a role in Redox-mediated Cortex Proliferation in the Arabidopsis Root — SPINDLY, ERECTA, and Its Ligand STOMAGEN Have a Role in Redox-Mediated Cortex Proliferation in the Arabidopsis Root — SPINDLY, ERECTA, and Its Ligand STOMAGEN Have a Role in Redox-Mediated Cortex Proliferation in the Arabidopsis Root — Supplementary Data 

# SPINDLY, ERECTA, and Its Ligand STOMAGEN Have a Role in Redox-Mediated Cortex Proliferation in the *Arabidopsis* Root

## Supplementary Data

Data files

**Files in this Data Supplement:**

- Supplementary Data - Supplementary Data
