# Supplementary material for: SPINDLY, ERECTA, and Its Ligand STOMAGEN Have a Role in Redox-Mediated Cortex Proliferation in the Arabidopsis Root
Source: Mol Plant. 2014 Sep 29;7(12):1727–39. doi: 10.1093/mp/ssu106 (PMC4261839; doi:10.1093/mp/ssu106)
Supplement: Supplementary Data [file supp_ssu106_Cui_et_al_suppl.pdf]

SUPPLEMENTARY FIGURE LEGENDS

Fig S1. Schematic illustration of the radial pattern and the position of the middle cortex in the Arabidopsis root.

Fig S2. Gene-ontology analysis of genes altered in *spy-3* root. Functional categorization was based on annotation for GO Biological Process.

Fig S3. GO enrichment analysis of genes altered by the *spy* mutation. The AmiGO program was used for this analysis. Shown: biological processes, molecular functions, and cellular compartments represented by the genes down-regulated (A–C) and up-regulated (D) in *spy-3* root.

Fig S4. The *ML1, IRX3* and *SUC2* promoters confer expression in the epidermis, xylem, and phloem, respectively in the Arabidopsis root. Shown: the GUS staining pattern in the roots of one-week-old transgenic plants expressing the GUS reporter gene under the control of these promoter sequences.

Fig S5. Confocal-microscopy images showing middle cortex in the roots of one-week-old root of *spy-12, spy-13, spy-15,* and *spy-17* seedlings. Framed areas are shown at the right at a higher magnification.

mc, middle cortex; c, cortex; e, endodermis. Bars = 20 μm.

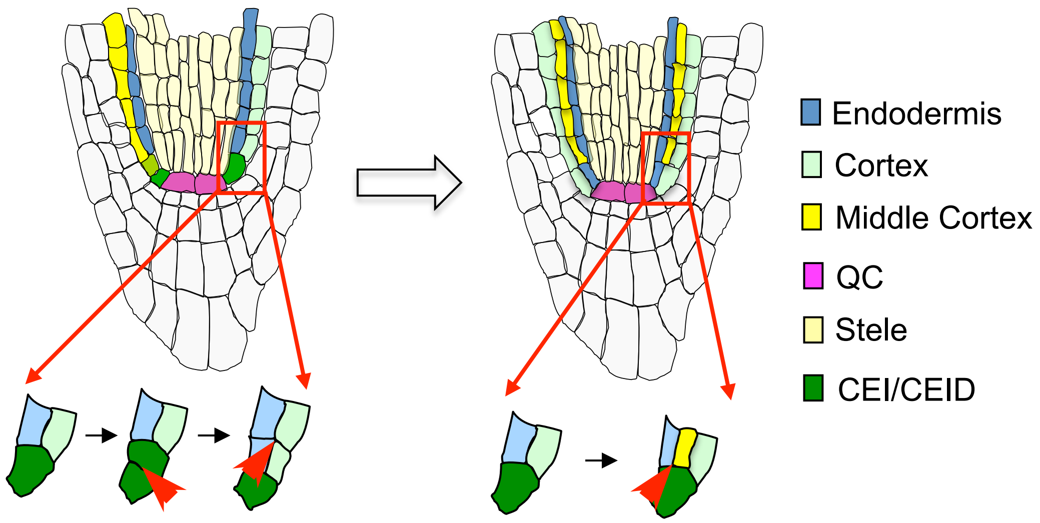

Fig S1. Schematic illustration of the radial pattern and the position of the middle cortex in the Arabidopsis root.

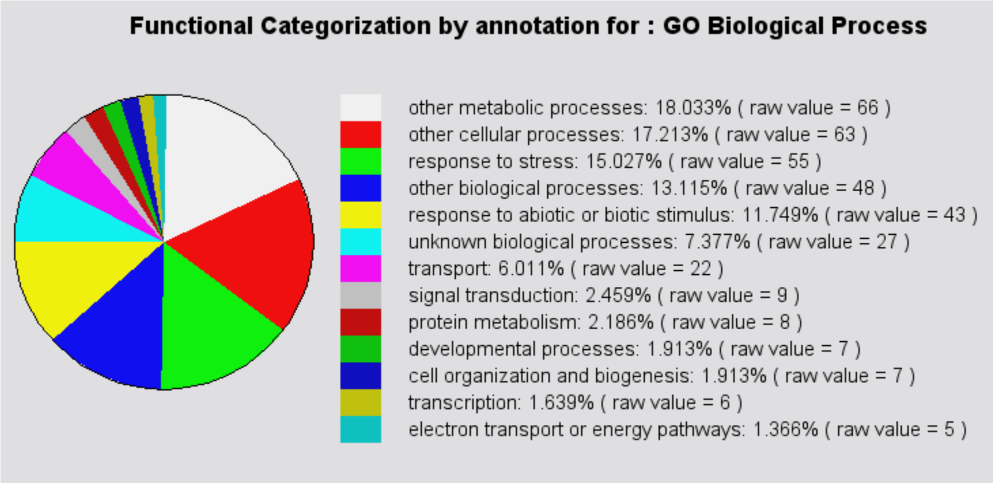

Fig S2. Gene-ontology analysis of genes altered in *spy-3* root. Functional categorization was based on annotation for GO Biological Process.

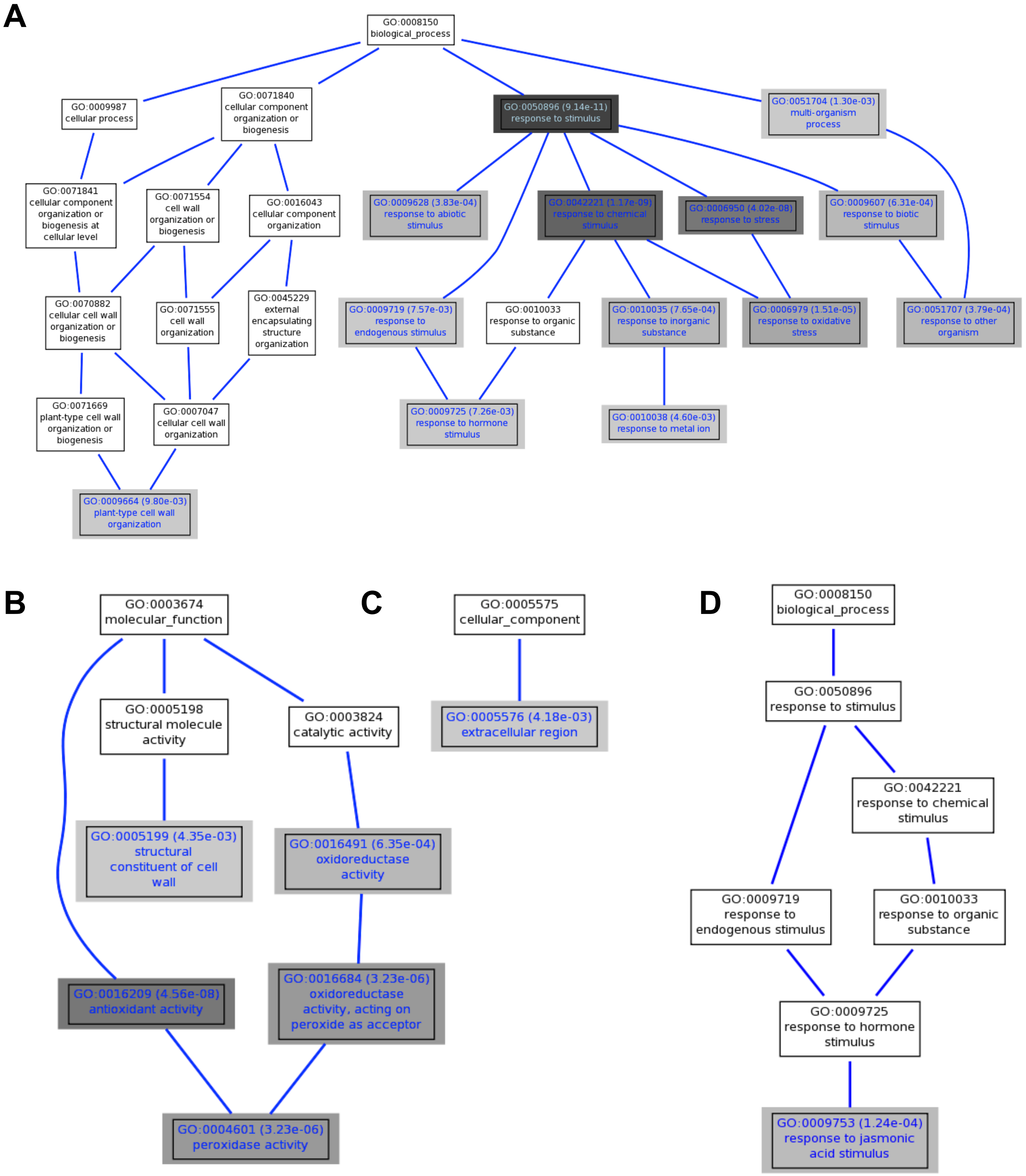

Fig S3. GO enrichment analysis of genes altered by the *spy* mutation. The AmiGO program was used for this analysis. Shown: biological processes, molecular functions, and cellular compartments represented by the genes down-regulated (A–C) and up-regulated (D) in *spy-3* root.

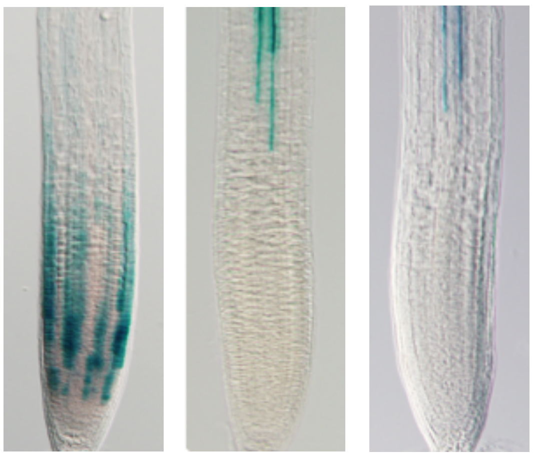

Fig S4. The *ML1, IRX3* and *SUC2* promoters confer expression in the epidermis, xylem, and phloem, respectively in the Arabidopsis root. Shown: the GUS staining pattern in the roots of one-week-old transgenic plants expressing the GUS reporter gene under the control of these promoter sequences.

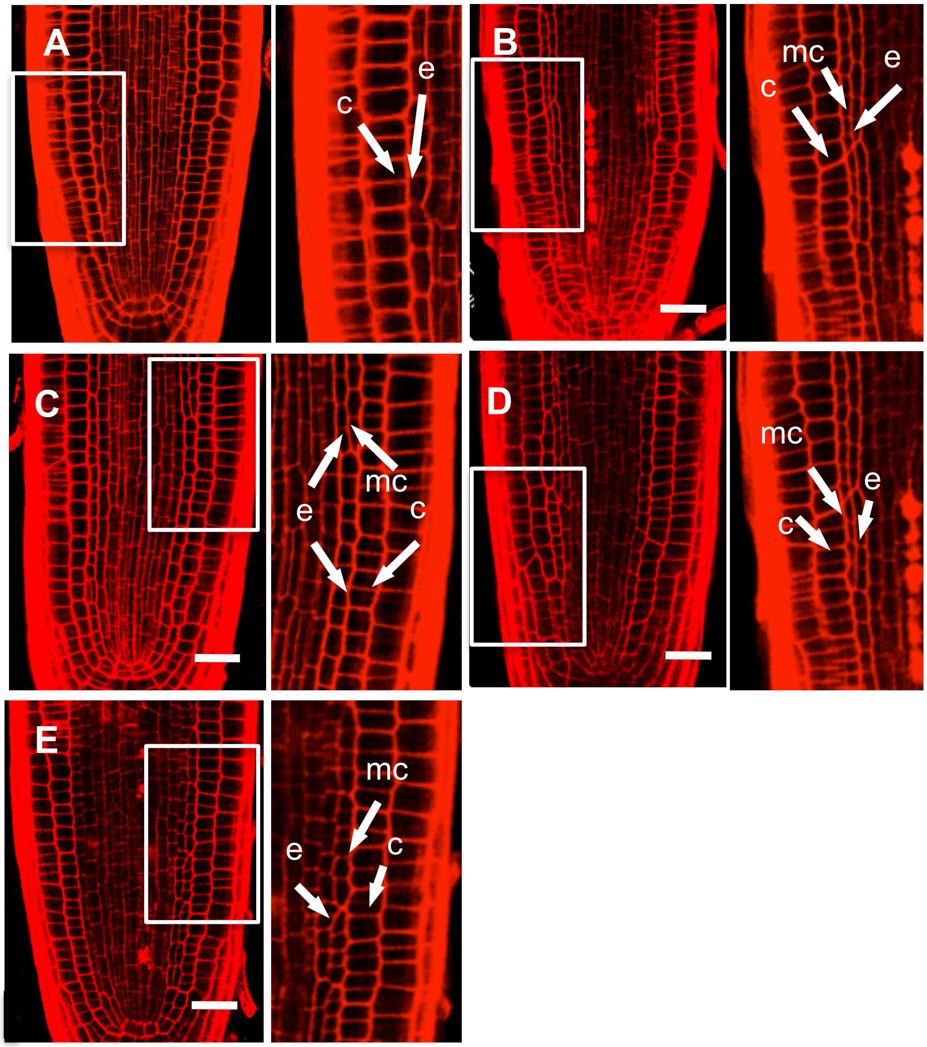

Fig S5. Confocal-microscopy images showing middle cortex in the roots of one-week-old root of Ler (A), *spy-12* (B)*, spy-13* (C)*, spy-15* (C)*,* and *spy-17* (E) seedlings. Framed areas are shown at the right at a higher magnification.

mc, middle cortex; c, cortex; e, endodermis. Bars = 20 μm.

Down regulated in *spy-3* root

PROBESET	QVALUE	Foldchange	WT_MEAN	SPY3_MEAN	GeneName
261567_at	6.2565E-19	5.21074422	4.48618229	0.860948474	AT1G33055
258487_at	2.0556E-18	3.14056473	3.31518071	1.055600186	AT3G02550
249675_at	5.6651E-20	3.07435263	4.96642246	1.615436827	AT5G35940
246114_at	4.6693E-18	2.84694016	18.167635	6.381460093	AT5G20250
250682_x_a	0.00043164	2.50589037	6.80653593	2.716214571	AT5G06630
251629_at	2.9279E-21	2.46214453	2.15705643	0.87608847	AT3G57410
253161_at	3.2001E-20	2.24675678	1.32038615	0.587685398	AT4G35770
254644_at	4.2591E-14	2.2066556	1.45350933	0.658693334	AT4G18510
254818_at	1.4356E-15	2.18447878	6.02344764	2.757384366	AT4G12470
252291_s_a	9.8869E-11	2.12291259	6.73392791	3.172023165	AT3G49120
258402_at	1.5808E-15	2.10848518	19.9610562	9.467012814	AT3G15450
249862_at	3.2719E-17	2.0435918	10.2065799	4.994431814	AT5G22920
258939_at	4.2244E-15	2.03082938	2.54940148	1.255349913	AT3G10020
246238_at	7.6778E-16	2.01657768	2.3538376	1.1672437	AT4G36670
260549_at	2.1526E-13	2.00977124	9.58533029	4.769363853	AT2G43535
246601_at	3.0297E-13	1.96182486	5.4959786	2.801462406	AT1G31710
260547_at	4.0044E-15	1.94173037	1.10900948	0.57114494	AT2G43550
258984_at	3.219E-18	1.9327736	1.63082477	0.843774339	AT3G08970
247540_at	2.5588E-17	1.91885546	3.86418822	2.01379849	AT5G61590
247327_at	2.0135E-11	1.9178884	16.2160562	8.45516151	AT5G64120
246195_at	1.1723E-17	1.9139341	1.47655563	0.771476735	AT4G36410
259264_at	1.0265E-12	1.90501216	2.09917078	1.101919887	AT3G01260
249337_at	8.2597E-06	1.87618477	3.97632054	2.119365108	AT5G41080
261157_at	1.317E-12	1.87009024	4.98429873	2.665271766	AT1G34510
252834_at	1.5666E-13	1.84806837	1.33297914	0.721282375	AT4G40070
252882_at	3.544E-07	1.84340925	4.90884345	2.662915707	AT4G39675
247293_at	2.1284E-19	1.83490179	1.16232131	0.633451507	AT5G64510
259544_at	1.8932E-19	1.81763864	2.32234049	1.277668966	AT1G20620
253332_at	3.1634E-10	1.81698386	2.99705807	1.649468735	AT4G33420
245668_at	8.707E-12	1.81297598	3.94194063	2.174292801	AT1G28330
257823_at	1.604E-11	1.81111933	3.88384875	2.144446627	AT3G25190
258225_at	2.7135E-08	1.80081842	6.15537358	3.418097849	AT3G15630
250868_at	1.9899E-21	1.79805486	1.70985127	0.95094499	AT5G03860
251109_at	1.6541E-14	1.79134026	8.23858243	4.599116423	AT5G01600
250683_x_a	1.5285E-06	1.7905013	4.84876373	2.708048151	AT5G06640
258256_at	0.00051441	1.75824569	2.57545081	1.464784375	AT3G26890
259181_at	2.4789E-15	1.75741706	3.94499272	2.244767518	AT3G01690
258930_at	3.3131E-08	1.74377431	0.85967222	0.492995117	AT3G10040
251438_s_a	6.6871E-11	1.73968508	15.3003795	8.794913341	AT3G59930;AT5G3335
255516_at	1.9571E-09	1.73451076	15.2905805	8.815500511	AT4G02270
259454_at	9.8339E-19	1.73359609	0.98843793	0.57016622	AT1G44050
249742_at	3.5384E-18	1.72615944	1.82909418	1.059632229	AT5G24490
261149_s_a	1.1198E-12	1.7240656	9.23322604	5.355495774	AT1G19570;AT1G1955
253666_at	3.6087E-11	1.71827354	3.61647235	2.104712821	AT4G30270
246375_at	5.1218E-12	1.71441709	2.83795838	1.655348862	AT1G51830
247871_at	9.1537E-13	1.70817576	5.04229049	2.951856953	AT5G57530
254225_at	0.00015222	1.70795463	11.1732393	6.541882967	AT4G23670
259076_at	3.4731E-09	1.70154189	1.64040966	0.96407245	AT3G02140
256255_at	6.2425E-13	1.70151645	3.77411059	2.21808645	AT3G11280
258421_at	5.5558E-07	1.69808685	2.76851005	1.630370113	AT3G16690
255886_at	4.6462E-13	1.69571255	1.99361939	1.175682393	AT1G20340
254092_at	4.3578E-22	1.69147073	1.63405976	0.966058551	AT4G25090
254534_at	8.9558E-15	1.68850893	2.54349606	1.506356296	AT4G19680
257924_at	1.1088E-13	1.68643335	3.74959542	2.223387848	AT3G23190
254907_at	1.9093E-09	1.6829452	6.27580076	3.729058304	AT4G11190

258434_at	6.8384E-14	1.68085198	1.31189569	0.780494477	AT3G16770
256774_at	2.7139E-08	1.66886528	2.22359979	1.332402216	AT3G13760
245637_at	6.5059E-05	1.66666155	4.01605044	2.409637655	AT1G25230
255127_at	2.2353E-16	1.65837171	2.50496198	1.510494883	AT4G08300
245136_at	8.6236E-14	1.65503129	1.5648362	0.945502486	AT2G45210
259980_at	4.8798E-19	1.65281436	1.98949729	1.203702815	AT1G76520
259383_at	1.165E-08	1.65140197	9.93438486	6.015727875	AT3G16470
255648_at	5.4272E-10	1.64922346	0.90077849	0.546183409	AT4G00910
259478_at	3.621E-05	1.64889245	6.31904978	3.832299538	AT1G18980
245925_at	1.4704E-16	1.63068416	1.94349792	1.19182976	AT5G28770
260693_at	1.4003E-09	1.62861841	11.2678609	6.918662373	AT1G32450
259982_at	1.3255E-14	1.628231	1.2234572	0.751402723	AT1G76410
255435_at	3.4009E-21	1.627958	1.22305219	0.751279939	AT4G03280
254805_at	6.5049E-09	1.62605899	1.22770633	0.755019549	AT4G12480
251842_at	4.4002E-11	1.6138777	3.008347	1.864048925	AT3G54580
260236_at	1.7209E-17	1.61256393	2.32214287	1.440031515	AT1G74470
251356_at	1.7319E-07	1.61046386	3.61772621	2.246387706	AT3G61060
254025_at	7.9287E-08	1.6071162	3.15980119	1.966131138	AT4G25790
259846_at	5.9763E-13	1.60386607	1.77160408	1.104583549	AT1G72140
260287_at	2.1906E-08	1.6022095	4.90836871	3.063499954	AT1G80440
252367_at	2.2119E-07	1.59977473	4.21770547	2.636437119	AT3G48360
253613_at	7.4748E-10	1.59570189	3.35262224	2.10103294	AT4G30320
252872_at	3.3772E-17	1.59569818	2.46502053	1.544791216	AT4G40010
257504_at	5.9292E-16	1.59466166	1.61500977	1.012760145	AT1G52250
254107_at	1.5792E-11	1.59418336	1.68679322	1.058092352	AT4G25220
251249_at	1.2641E-17	1.59364605	1.24868328	0.783538657	AT3G62160
260741_at	1.1799E-07	1.59326487	5.06637651	3.179870844	AT1G15040
247280_at	1.0015E-07	1.57904353	13.9960425	8.863620443	AT5G64260
253687_at	5.9592E-06	1.57755103	4.04876448	2.566487177	AT4G29520
257315_at	1.0684E-12	1.57734717	9.67034954	6.130767983	AT3G30775
254550_at	1.1861E-05	1.57011039	6.86360095	4.371412983	AT4G19690
250474_at	3.983E-09	1.5639956	2.42509007	1.55057346	AT5G10230
260284_at	5.5665E-06	1.56300545	5.96903438	3.818946617	AT1G80380
246991_at	3.5892E-10	1.55602332	13.0480213	8.385492153	AT5G67400
248828_at	1.2452E-11	1.55596796	2.287607	1.470214721	AT5G47110
251443_at	6.9806E-16	1.55230379	1.53351895	0.98789874	AT3G59940
259609_at	4.3605E-09	1.55209054	0.80898513	0.521222897	AT1G52410
246595_at	1.4485E-14	1.55014004	1.59309634	1.027711239	AT5G14780
253163_at	9.9113E-07	1.54534798	9.4803271	6.134752314	AT4G35750
248050_at	1.5348E-13	1.54079135	1.18367709	0.768226724	AT5G56100
247333_at	2.0431E-05	1.53286411	10.3594584	6.758236626	AT5G63600
259841_at	5.383E-05	1.52937817	12.0340031	7.868559481	AT1G52200
259757_at	0.00013168	1.52618728	7.04960232	4.619093881	AT1G77510
255290_at	1.2058E-08	1.52313807	2.3685209	1.555027049	AT4G04640
258527_at	5.1756E-09	1.51271457	2.88141581	1.904798084	AT3G06850
247813_at	1.2867E-13	1.50974787	1.19213788	0.789627136	AT5G58330
253228_at	8.1575E-07	1.50918768	4.91112513	3.254151344	AT4G34630
248371_at	1.0563E-11	1.50706731	0.86751592	0.575631834	AT5G51810
256302_at	6.3472E-14	1.50678271	3.89709591	2.586368879	AT1G69526
249800_at	1.3153E-13	1.50273849	2.2214007	1.478235043	AT5G23660
255140_x_a	0.00024917	1.50136937	4.31864959	2.876473753	AT4G08410

description
Expressed protein
LOB domain protein 41 / lateral organ boundaries domain protein 41 (LBD41),
Jacalin lectin family protein, similar to myrosinase-binding protein homolog
Similar to alkaline alpha galactosidase, putative [Arabidopsis thaliana]
Proline-rich extensin-like family protein, contains proline-rich extensin domains,
Villin 3 (VLN3), nearly identical to villin 3 (VLN3) (Arabidopsis thaliana) GI:3415117
Senescence associated protein (SEN1), Dark inducible 1 (DIN1). Pi-deficiency
Clavata3 / ESR-Related-2 (CLE2), CLAVATA3/ESR-Related-2 (CLE2)
Protease inhibitor/seed storage/lipid transfer protein (LTP) family protein, similar
AT3G49120, peroxidase 34; AT3G49110, Perx33
Expressed protein, similar to auxin down-regulated protein ARG10 (Vigna radiata)
Zinc finger (C3HC4-type RING finger) family protein, contains Pfam
Expressed protein
Mannitol transporter, putative, similar to mannitol transporter (Apium graveolens
Encodes a defensin-like (DEFL) family protein.
Copper amine oxidase, putative, similar to copper amine oxidase (Lens culinaris)
Encodes a defensin-like (DEFL) family protein.
DNAJ heat shock N-terminal domain-containing protein, low similarity to
Encodes a member of the ERF (ethylene response factor) subfamily B-3 of ERF/AP2
Peroxidase, putative, identical to peroxidase (Arabidopsis thaliana)
Ubiquitin-conjugating enzyme 17 (UBC17), E2; identical to gi:2801446
Aldose 1-epimerase family protein, similar to non-cell-autonomous protein
Glycerophosphoryl diester phosphodiesterase family protein, weak similarity to
Peroxidase, putative, similar to peroxidase ATP13a GB:CAA67312 from
Similar to zinc finger (C3HC4-type RING finger) family protein (ATL6) [Arabidopsis
Expressed protein
Expressed protein
Catalase 3 (SEN2), almost identical to catalase 3 SP:Q42547, GI:3123188 from
Peroxidase, putative, identical to class III peroxidase ATP32 (Arabidopsis thaliana)
Dormancy-associated protein, putative (DRM1), identical to dormancy-associated
Nodulin, putative, similar to nodulin 21 (N-21) (Glycine max) SWISS-PROT:P16313
Expressed protein
Malate synthase, putative, strong similarity to glyoxysomal malate synthase from
Ferritin 1 (FER1), identical to ferritin (Arabidopsis thaliana) GI:1246401,
Proline-rich extensin-like family protein, contains proline-rich extensin domains,
Expressed protein
Expressed protein
Expressed protein, est match
[AT3G59930, Encodes a defensin-like (DEFL) family protein.];[AT5G33355,
Pollen Ole e 1 allergen and extensin family protein, contains Pfam domain,
DC1 domain-containing protein, contains Pfam profile PF03107: DC1 domain
30S ribosomal protein, putative, similar to SP:P19954 Plastid-specific 30S
Both genes encodes dehydroascorbate reductase
MERI-5 protein (MERI-5) (MERI5B) / endo-xyloglucan transferase / xyloglucan
Leucine-rich repeat protein kinase, putative, similar to light repressible receptor
Xyloglucan:xyloglucosyl transferase, putative / xyloglucan endotransglycosylase,
Major latex protein-related / MLP-related, low similarity to major latex protein
Expressed protein
Myb family transcription factor, contains Pfam profile: PF00249 Myb-like DNA-
Nodulin MtN3 family protein, contains Pfam PF03083 MtN3/saliva family
Plastocyanin, similar to plastocyanin GI:1865683 from (Arabidopsis thaliana)
RBOHD, respiratory burst oxidase, putative / NADPH oxidase
Iron-responsive transporter (IRT2), member of the Zinc (Zn2+)-Iron (Fe2+)
Lesion inducing protein-related, similar to ORF, able to induce HR-like lesions
Disease resistance-responsive family protein / dirigent family protein, similar to

ERF72, the ERF (ethylene response factor) subfamily B-2 of ERF/AP2 transcription
DC1 domain-containing protein, contains Pfam profile PF03107: DC1 domain
Purple acid phosphatase family protein, contains Pfam profile: PF00149 calcineurin-
Nodulin MtN21 family protein, similar to MtN21 GI:2598575 (root nodule
Auxin-responsive protein-related, weakly similar to small auxin up RNA
Auxin efflux carrier family protein, contains auxin efflux carrier domain,
Jacalin lectin family protein, contains Pfam profile: PF01419 jacalin-like lectin
Expressed protein, contains Pfam profile PF01027: Uncharacterized protein family
Germin-like protein, putative, similar to germin-like protein subfamily T member 1
bZIP transcription factor family protein, similar to seed storage protein opaque-
Proton-dependent oligopeptide transport (POT) family protein, contains Pfam
Zinc finger (C3HC4-type RING finger) family protein, contains Pfam domain,
Cytochrome B6-F complex iron-sulfur subunit, chloroplast / Rieske iron-sulfur
Protease inhibitor/seed storage/lipid transfer protein (LTP) family protein,
Proline-rich extensin-like family protein, contains proline-rich extensin domains,
Geranylgeranyl reductase, identical to geranylgeranyl reductase GB:Y14044
F-box family protein / lectin-related, low similarity to PP2 lectin polypeptide
Allergen V5/Tpx-1-related family protein, similar to SP:Q40374 Pathogenesis-
Proton-dependent oligopeptide transport (POT) family protein, contains Pfam
Kelch repeat-containing F-box family protein, similar to SP:Q9ER30 Kelch-related
Speckle-type POZ protein-related, contains Pfam PF00651 : BTB/POZ domain;
Allergen V5/Tpx-1-related family protein, similar to SP:Q40374 Pathogenesis-
Serine/threonine protein kinase, putative, similar to serine-threonine protein
Dynein light chain type 1 family protein, similar to SP:O02414 Dynein light chain
Transporter, putative, similar to glycerol-3-phosphate transporter (glycerol 3-
Transferase family protein, low similarity to Taxus cuspidata transferases: 10-
Glutamine amidotransferase-related
Phosphate-responsive protein, putative, similar to phi-1 (phosphate-induced gene)
Expressed protein
Proline oxidase, mitochondrial / osmotic stress-responsive proline dehydrogenase
Iron-responsive transporter (IRT1), identical to Fe(II) transport protein
Annexin 7 (ANN7), nearly identical to calcium-binding protein annexin 7
Phosphoribulokinase/uridine kinase-related
Peroxidase 73 (PER73) (P73) (PRXR11), identical to SP:Q43873 Peroxidase 73
Lil3 protein, putative, similar to Lil3 protein (Arabidopsis thaliana)
Kelch repeat-containing F-box family protein, contains Pfam profiles PF01344:
Caldesmon-related, weak similarity to Caldesmon (CDM) (Swiss-Prot:P12957)
Formate dehydrogenase (FDH), identical to GI:7677266
Rho-GTPase-activating protein-related, contains weak similarity to Rho-GTPase-
Glycine-rich protein / oleosin, contains Pfam PF01277: Oleosin domain
Flavonol synthase, putative, similar to SP:Q96330; contains Pfam profile PF03171:
Expressed protein, similar to PGPS/D12 (Petunia x hybrida) GI:4105794; contains
Encodes a protein disulfide isomerase-like (PDIL) protein, a member of a multigene
ATP synthase gamma chain 1, chloroplast (ATPC1), identical to SP:Q01908 ATP
Branched chain alpha-keto acid dehydrogenase E2 subunit (din3), identical to
Malate dehydrogenase (NADP), chloroplast, putative, strong similiarity to
Expressed protein
Encodes gibberellin 20-oxidase. Involved in gibberellin biosynthesis. Up-regulated
UbiE/COQ5 methyltransferase family protein, low similarity to SP:Q05197
Nodulin MtN3 family protein, similar to MtN3 GI:1619602 (root nodule
Proline-rich extensin-like family protein, contains proline-rich extensin domains,

Upregulated in *spy-3* root

PROBESET	QVALUE	Foldchange	WT_MEAN	SPY3_MEAN	GeneName
258321_at	1.576E-16	2.2894982	1.2692223	2.90588233	AT3G22840
247463_at	9.073E-13	2.1936634	1.7469028	3.83211676	AT5G62210
245928_s_	4.139E-18	2.0299978	0.7396035	1.50139345	AT5G24770;AT
253259_at	1.605E-18	2.0141501	0.6991625	1.40821826	AT4G34410
261443_at	3.935E-12	1.9784271	1.1412976	2.25797419	AT1G28480
258217_at	4.732E-14	1.9688663	3.6920107	7.26907537	AT3G18000
245341_at	2.235E-16	1.9373447	1.0377818	2.01054112	AT4G16447
248794_at	1.886E-19	1.8972664	0.5366021	1.0180771	AT5G47220
245306_at	4.88E-19	1.8789241	1.5446547	2.90228894	AT4G14690
249894_at	1.091E-15	1.8665719	0.5937052	1.10819338	AT5G22580
246888_at	1.625E-09	1.8537551	2.05847	3.81589916	AT5G26270
246814_at	1.481E-17	1.8507825	0.6185703	1.14483901	AT5G27200
258145_at	3.49E-10	1.6821494	3.3032814	5.55661302	AT3G18200
260067_at	5.227E-16	1.6737381	1.2876934	2.15526148	AT1G73780
258218_at	2.1E-07	1.6681322	5.0205699	8.37497415	AT3G18000
248048_at	1.471E-11	1.6450317	1.1479185	1.88836222	AT5G56080
254158_at	2.161E-05	1.631901	1.8252195	2.97857758	AT4G24380
252988_at	1.057E-20	1.6253982	1.196362	1.94456466	AT4G38410
245041_at	3.291E-05	1.6108129	3.0616733	4.93178279	AT2G26530
260429_at	2.428E-10	1.5590744	1.4134724	2.20370868	AT1G72450
254524_at	8.666E-14	1.5407044	0.8959236	1.38035342	AT4G20000
247625_at	2.629E-09	1.5386136	1.2171215	1.87267976	AT5G60200
245560_at	5.917E-16	1.5383141	0.7442175	1.14484029	AT4G15480
245185_at	8.401E-15	1.5302292	0.933897	1.42907639	AT1G67760
252327_at	6.563E-08	1.5259786	2.4429465	3.72788391	AT3G48740
257536_at	1.721E-17	1.5072734	0.8948766	1.34882369	AT3G02800

description
Chlorophyll A-B binding family protein / early light-induced protein (ELIP),
Embryo-specific protein-related, contains weak similarity to embryo-specific
[AT5G24770, vegetative storage protein 2 (VSP2), identical to SP:O82122
RRTF1, ERF (ethylene response factor) subfamily B-3 of ERF/AP2 transcription
Glutaredoxin family protein, contains INTERPRO Domain IPR002109,
Phosphoethanolamine N-methyltransferase 1 / PEAMT 1 (NMT1), identical to
Expressed protein
Encodes a member of the ERF (ethylene response factor) subfamily B-3 of
Chlorophyll A-B binding family protein / early light-induced protein, putative,
Expressed protein
Expressed protein, ; expression supported by MPSS
Acyl carrier protein, chloroplast, putative / ACP, putative, similar to Acyl carrier
Nodulin MtN21 family protein, similar to MtN21 GI:2598575 (root nodule
Protease inhibitor/seed storage/lipid transfer protein (LTP) family protein,
Phosphoethanolamine N-methyltransferase 1 / PEAMT 1 (NMT1), identical to
Nicotianamine synthase, putative, similar to nicotianamine synthase
Expressed protein, contains Pfam profile: PF03959 domain of unknown function
Dehydrin, putative, similar to dehydrin ERD10 (Low-temperature-induced protein
Expressed protein
Expressed protein
VQ motif-containing protein, contains PF05678: VQ motif
Dof-type zinc finger domain-containing protein, similar to dof6 zinc finger protein
UDP-glucoronosyl/UDP-glucosyl transferase family protein, contains Pfam
Similar to T-complex protein 1 epsilon subunit, putative / TCP-1-epsilon, putative
Nodulin MtN3 family protein, similar to MtN3 GI:1619602 (root nodule
Similar to tyrosine specific protein phosphatase family protein [Arabidopsis
